# Prognostic Value of HPV E6 and APOBEC3B in Upper Urinary Tract Urothelial Carcinoma

**DOI:** 10.1155/2022/2147494

**Published:** 2022-07-19

**Authors:** Le He, Bo Yang, Dan Jian, Hao Luo, Dong Wang, Nan Dai

**Affiliations:** ^1^Cancer Center, Daping Hospital & Army Medical Center of PLA, Third Military Medical University (Army Medical University), Chongqing, China; ^2^Department of Gastroenterology, Chongqing General Hospital, Chongqing, China

## Abstract

**Background:**

APOBEC mutation signature is common in upper urinary tract urothelial carcinoma (UTUC). When virus infection occurs, upregulated APOBEC plays an antiviral role by deoxycytidine deaminase activity. However, the carcinogenic roles of HPV E6 protein and APOBEC mutation signature in UTUC have not been investigated.

**Aims:**

This study explored the relationship among HPV E6, APOBEC, and clinicopathological characteristics in patients with UTUC and impacts of their expression on the prognosis.

**Methods:**

The expression of HPV E6 and APOBEC3B of 78 patients with UTUC was detected by immunohistochemistry. Correlation of HPV E6 and APOBEC3B expression levels with clinicopathological characteristics was statistically analyzed. Univariate and multivariate Cox regression analyses were used to evaluate the prognosis of HPV E6 and APOBEC3B for disease-free survival (DFS); survival analysis was performed using Kaplan-Meier methods.

**Results:**

The expression of APOBEC3B was positively correlated with the expression of HPV E6 (*r* = 0.383, *P* = 0.001). HPV E6 was significantly increased in patients with stage I (*χ*^2^ = 4.938, *P* = 0.026) and low-grade urothelial carcinoma (*χ*^2^ = 3.939, *P* = 0.047), as well as in patients without LVI (*χ*^2^ = 4.064, *P* = 0.044). Meanwhile, APOBEC3B was highly expressed in patients with stage I (*χ*^2^ = 4.057, *P* = 0.044) and low-grade urothelial carcinoma (*χ*^2^ = 7.153, *P* = 0.007). Multivariate Cox regression analysis revealed the APOBEC3B expression was the independent prognostic factor for DFS, Kaplan-Meier survival analysis showed that low expression of APOBEC3B and HPV E6 was significantly associated with the poor prognosis of UTUC patients.

**Conclusion:**

HPV E6 expression is positively associated with APOBEC3B expression, and the high expression of HPV E6 and APOBEC3B is associated with favorable prognosis of patients with UTUC.

## 1. Introduction

Upper urinary tract urothelial carcinoma (UTUC) refers to urothelial carcinoma that occurs in the ureter and renal pelvis, accounting for 5-10% of all urothelial carcinomas [1]. Although the pathological origin of UTUC is the same as that of bladder cancer and the pathogenesis is similar, UTUC has a worse prognosis. It is currently believed that the pathogenesis of urothelial carcinoma is related to the exposure of aromatic chemicals and tobacco exposure [2, 3] and the intake of certain Chinese herbal medicines containing aristolochic acid [4]. Recent studies on bladder cancer have found that human papillomavirus (HPV) infection is closely related to carcinogenesis, squamous differentiation, and recurrence of bladder cancer [5–7]. However, the crucial role of HPV in the carcinogenesis of UTUC remains unclear.

Apolipoprotein B mRNA-editing enzyme catalytic polypeptide (APOBEC) family members are evolutionarily conserved cytidine deaminases, which play an important role in human innate and acquired immune mechanisms, such as regulating the activation of inflammatory cytokines and chemokines [8]. Among them, APOBEC3A and APOBEC3B are the two most important enzymes. APOBEC single-base substitution mutation (APOBEC signature) is characterized by C-to-T and C-to-G mutations in the 5′-TC(A/T) trinucleotide motif [9, 10]. Although this mechanism can inhibit the viral replication to achieve antiviral effects, it can also cause single-stranded DNA and/or RNA point mutations in the host genome during replication, transcription, or translation. The accumulation of these point mutations will provide a genetic basis for tumorigenesis [8, 11]. Although APOBEC3B has been found to be upregulated in a variety of tumors, the correlation between APOBEC3B signature and HPV carcinogenesis remains unclear. The carcinogenic effects of HPV in cervical cancer and head and neck squamous cell carcinoma have been extensively studied. The proteins E6 and E7 encoded by high-risk HPV16 and HPV18 have been found to promote tumorigenesis [12]. Several studies have found that E6 can directly participate in the transcriptional upregulation of APOBEC3B [10, 13] and contribute to APOBEC3B mutation signature, which is widely found in a variety of tumors, including cervical cancer, head and neck squamous cell carcinoma, bladder cancer, and lung cancer [8, 14]. A study suggested that of the most common FGFR3 mutation in about 65% of bladder cancer is caused by APOBEC, and APOBEC mutation signature plays an important biological role in bladder cancer [15]. Although the overall mutation load of UTUC is lower than that of bladder cancer, the APOBEC mutation signature in UTUC is the most common, accounting for 45% of all mutation signature [16]. In this study, we hypothesized that HPV E6 protein can upregulate the expression of APOBEC3B, which causes a large number of mutations in the urothelial epithelium and promotes carcinogenesis. The purpose of this study is to examine the correlation between expression of E6 and APOBEC3B in UTUC by immunohistochemical analysis and evaluate their relationship with clinicopathological characteristics and prognosis. The results will provide clinical evidence for basic research.

## 2. Materials and Methods

### 2.1. Research Design and Patients

From January 2014 to January 2017, a total of 85 patients were histologically confirmed as primary UTUC, and they did not receive any preoperative treatment before undergoing radical surgery at the Army Medical Center of Third Military Medical University. Clinical staging was based on the AJCC staging method for urothelial carcinoma. Among them, 5 cases were lost to follow-up, and 2 cases were unqualified samples. Therefore, 78 patients were eventually included in this study, and the clinical characteristics of the patients were obtained from the hospital medical record system. Follow-up cystoscopy was performed every 3 months for the first 2 years, every 6 months for the next 2 years, and then, annually. Other follow-up examinations included physical examination, urinary cytology, and chest and abdominal computed tomography (CT). In addition, a telephone follow-up was conducted to determine whether the tumor had recurred or whether the patient is still alive. The study was approved by the Ethics Review Committee of the Army Medical Center and was performed in accordance with the Declaration of Helsinki.

Disease-free survival (DFS) was measured from the date of the initial treatment to the date of diagnosis of locoregional recurrence or distant metastasis.

### 2.2. Immunohistochemical Analysis of Tissue Microarray

Tissue microarrays (TMA) containing 78 patients' tissues were used for subsequent immunohistochemistry (IHC) to examine the expression of E6 and APOBEC3B. Consecutive 4 *μ*m thick sections were cut from a TMA block. The tissue sections were incubated at 60°C for 6 h, deparaffinized in xylene, and then, rehydrated in declining ethanol dilutions. For heat-induced epitope retrieval (HIER), the citric acid thermal repair was performed according to the antibody instructions. Subsequently, the slides were cooled to room temperature, and the endogenous peroxidase activity was blocked with 0.3% hydrogen peroxide (Merck Millipore) in PBS for 10 minutes at room temperature. The slide sections were incubated with primary antibody E6 (Abcam, #Ab70; 1 : 200 dilution) or APOBEC3B (Abcam, #Ab191695; 1 : 200 dilution) in a humidified chamber at 4°C overnight. Afterwards, the slide sections were incubated with biotinylated rabbit anti-mouse IgG/anti-rabbit IgG secondary antibody at 37°C for 30 minutes. The signal was visualized using the chromogenic substrate diaminobenzidine (DAB) and counterstained with hematoxylin.

The IHC slides were scored independently by two pathologists, who blinded to the patients' clinical outcomes. If the scoring results of the two pathologists are inconsistent, the slides are reevaluated under a multihead microscope, and the final score will be determined through discussion. The expression score of APOBEC3B by IHC was calculated by multiplying the score of staining intensity by that of the percentage of positive cells. IHC staining intensity score was stratified from 0 to 3 (0: no staining; 1: mild staining; 2: moderate staining; and 3: intense staining). The percentage of positive cells was scored as follows: 1, the percent of stained cells <1/3; 2, 1/3 ≤ the percent of stained cells < 2/3; 3, the percent of stained cells ≥2/3. Finally, the expression of APOBEC3B was sorted into low expression (scores 0-4) and high expression (scores 6, 9). HPV E6 was rated as positive or negative according to whether or not it was expressed.

Both negative control and positive control of APOBEC3B and E6 expression were also stained according to the introduction of antibody.

### 2.3. Statistical Analysis

Statistical analysis was performed using SPSS software version 21. Chi-square test was used to compare the rates of the two groups. Kaplan-Meier method was used to estimate cumulative survival probability, and log-rank test was used to examine the significance of survival difference between groups. A multivariate analysis was performed using the Cox proportional hazard model. All tests were two-sided with a 95% confidence interval (CI), and differences with *P* < 0.05 were considered as statistically significant. For multiple comparisons, the *P* value was adjusted according to the Benjamin and Hochberg's method.

## 3. Result

### 3.1. Demographics and Clinicopathological Characteristics

A total of 70 patients underwent radical nephroureterectomy with bladder cuff resection. The other 5 and 3 patients underwent nephrectomy and ureterectomy, respectively. All patients received intravesical chemotherapy after surgery, and eight patients received platinum-based adjuvant chemotherapy. The median follow-up time was 18 months (range 2-60 months). The demographic characteristics of the patients are shown in Table 1. The male-to-female ratio in the study population was 2 : 1. Among the patients, 33 had a history of smoking, accounting for 42.31%. The location of primary tumors of 47 (60.26%) and 31 (39.74%) patients located in renal pelvic and ureter, respectively. Fourteen patients were diagnosed with low-grade urothelial carcinomas, while sixty-four patients were diagnosed as high-grade urothelial carcinomas. In addition, 33 cases were nonmuscle-invasive tumors (pTa or pT1), and 45 cases were muscle-invasive tumors (pT2, pT3, or pT4). Lymphovascular invasion (LVI) was found in 16 of 78 patients (20.51%). There were 73 patients with pNx or pN0 stage, 5 patients with pN+ stage, and no patients with distant metastases.

### 3.2. APOBEC3B and E6 Expression Levels Are Associated with Clinicopathology

**Figure 1:**
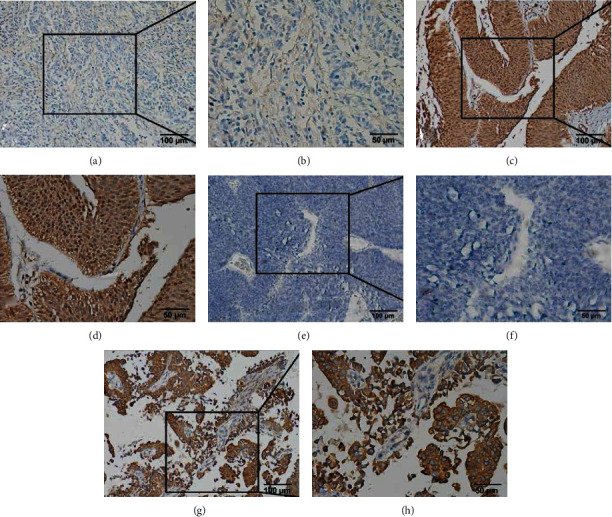
Representative immunohistochemical staining microphotographs of E6 and APOBEC3B expression in UTUC. (a, b) The low expression of APOBEC3B, respectively. (c, d) The high expression of APOBEC3B, respectively. (e, f) Negative expression of E6, respectively. (g, h) The positive expression of E6, respectively.

**Figure 2:**
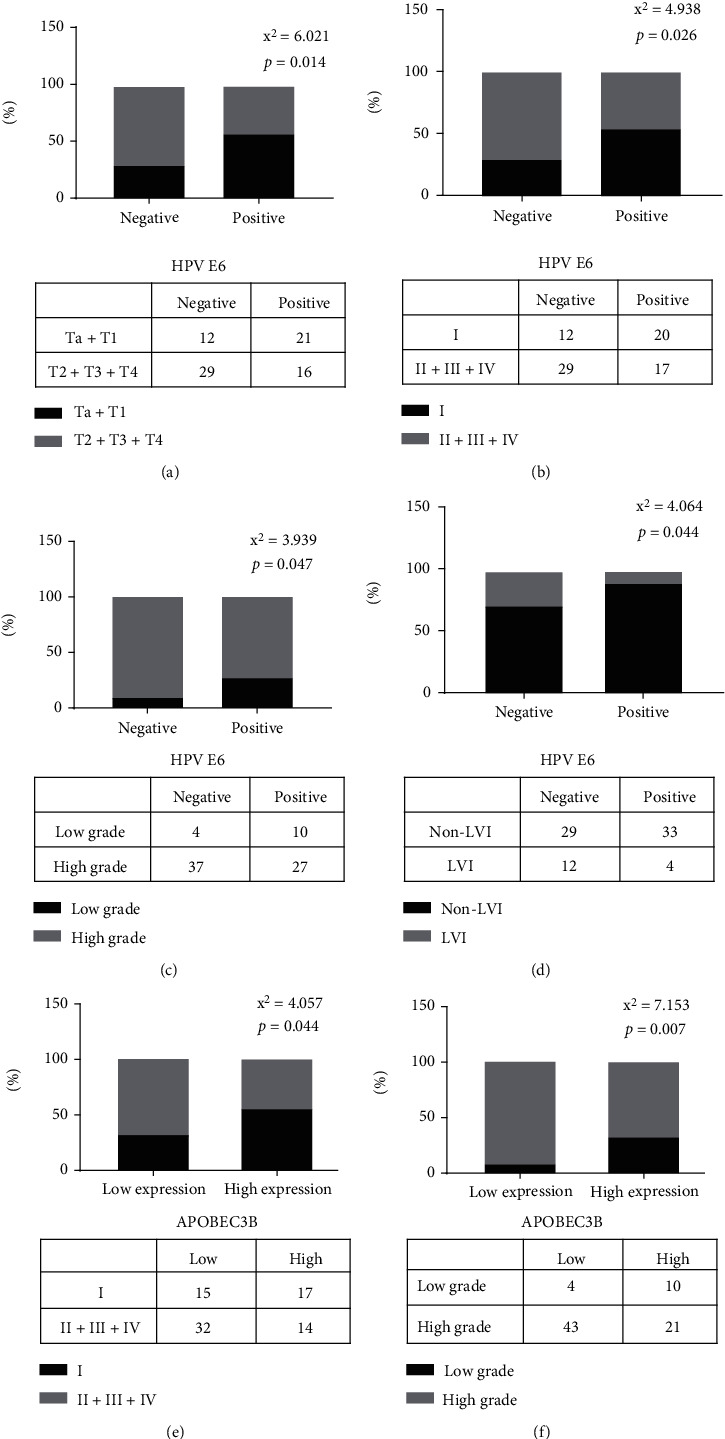
Chi-square test to analyze the relations between APOBEC3B, HPV E6 expression, and clinical features. The relations between HPV E6 expression and stag e (a), AJCC stage (b), grade (c), LVI (d); the relations between APOBEC3B expression and AJCC stage (e) and grade (f).

The results of IHC analysis showed that APOBEC3B protein expression was upregulated in UTUC tumor tissues, with a positive rate of 93.59% (73 out of 78 tissues). APOBEC3B was mainly expressed in the nucleus and moderately expressed in cytoplasm, while E6 protein expression was mainly expressed in cytoplasm (Figure 1). There were 37 cases with E6 protein positive, with a positive rate of 47.44%. There were 22 cases with high expression of AOPBEC3B and positive expression of E6. The expression of APOBEC3B and E6 was compared in terms of pathological T stage, pathological N stage, tumor grade, and LVI by linear regression analysis. E6 expression was significantly correlated with tumor stage and tumor grade. E6 protein was significantly expressed in patients with pathological Ta and T1 stage (*χ*^2^ = 6.021, *P* = 0.014), stage I (*χ*^2^ = 4.938, *P* = 0.026), and low-grade urothelial carcinoma (*χ*^2^ = 3.939, *P* = 0.047), as well as patients without LVI (*χ*^2^ = 4.064, *P* = 0.044). In addition, APOBEC3B was significantly highly expressed in patients with stage I (*χ*^2^ = 4.057, *P* = 0.044) and low grade (*χ*^2^ = 7.153, *P* = 0.007) (Figure 2).

### 3.3. Higher APOBEC3B and E6 Expression Is Associated with Favorable Prognosis in UTUC Patients

**Figure 3:**
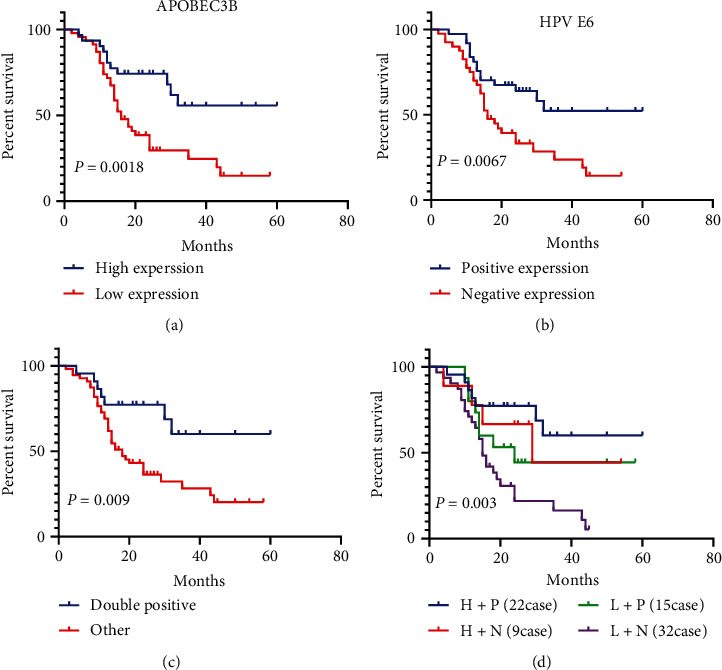
Kaplan-Meier survival analysis of HPV E6 and APOBEC3B with DFS of patients with UTUC. (a) Effect of the APOBEC3B expression on DFS; (b) effect of the HPV E6 expression on DFS; (c) effect of the APOBEC3B and HPV E6 double-positive expression on DFS; (d) effect of the different expression patterns of APOBEC3B and HPV E6 expression on DFS.

Kaplan-Meier survival analysis was used to appraise the prognostic value of APOBEC3B and E6 expression in UTUC patients. The results showed that poor DFS was significantly associated with low APOBEC3B expression (Figure 3(a), *P* = 0.0018) and negative E6 expression (Figure 3(b), *P* = 0.0067). Among these patients, 22 patients (28.21%) had both high expression of AOPBEC3B and positive expression E6. Kaplan-Meier survival analysis showed that patients with both high AOPBEC3B and positive E6 expression significantly associated with better DFS than patients with other expression levels (Figure 3(c), *P* = 0.009). Kaplan-Meier survival analysis of the four groups (according to the expression of APOBEC3B and E6) showed that the high expression of APOBEC3B and the positive expression of E6 had the best prognosis (Figure 3(d), *P* = 0.003).

Univariate and multivariate Cox regression analyses were conducted to examine the association between survival outcomes and clinicopathological characteristics and the expression of APOBEC3B and E6 (Table 2). Results of univariate analysis showed that DFS was significantly associated with the expression APOBEC3B (HR: 0.348, 95%CI 0.176-0.690, *P* = 0.002) and E6 (HR: 0.426, 95%CI 0.230-0.792, *P* = 0.007), LVI (HR: 2.103, 95%CI 1.102-4.015, *P* = 0.024), T staging (HR: 2.346, 95%CI 1.242-4.431, *P* = 0.009), and N staging (HR: 3.241, 95%CI 1.144-9.182, *P* = 0.027). Multivariate Cox regression analysis further revealed that APOBEC3B expression was the only independent prognostic factor for DFS (HR: 0.408, 95%CI 0.203-0.821, *P* = 0.012).

### 3.4. The Correlation between the Expression of APOBEC3B and E6 with Clinicopathological Characteristics

Pearson's coefficient correlation analysis was conducted to examine the correlation between APOBEC3B expression, E6 expression, and clinicopathological characteristics (Table 3). The results showed that APOBEC3B expression was positively correlated with E6 expression (*r* = 0.383, *P* = 0.001), but was negatively correlated with T staging (*r* = −0.251, *P* = 0.028). In addition, HPV E6 was negatively correlated with T stage (*r* = −0.225, *P* = 0.048) and was negatively correlated with LVI (*r* = −0.228, *P* = 0.045). There was a positive correlation between T stage and LVI (*r* = 0.413, *P* < 0.001).

## 4. Discussion

UTUC is a rare type of urothelial carcinoma. The pathogenesis of UTUC is currently believed to be related to the intake of benzene ring chemicals from tobacco and aristolochic acid. Although there is still a lack of consensus, several studies have reported that HPV infection is related to the occurrence of urothelial carcinoma [5, 17–20]. HPV infection is related to the tumorigenesis of cervical cancer and head and neck squamous cell carcinoma. In addition, HPV infection upregulates the expression of APOBEC, thereby promoting the instability of host genome and leading to the tumorigenesis. Furthermore, APOBEC mutation signature is the main feature of urothelial cancer genomes. For example, FGFR3 S249C mutation is one of the typical APOBEC mutation signature, accounting for 60% of all FGFR3 mutation profiles in the bladder cancer and UTUC [15, 16]. Another study of 9 patients with cervical cancer found that all of these patients had FGFR3 S249C mutations [15], strongly suggesting that HPV may play an important role in the development of urothelial carcinoma.

Many studies have reported the positive rate of HPV infection in bladder cancer, and PCR is the most common method for detecting HPV infection in tumor tissues [5, 7, 21–24]. Overall, the positive rate of HPV infection in bladder cancer is around 15%. A recent study based on Chinese population found that the HPV infection rate in bladder cancer was 28.77% [25]. Some studies revealed the positive correlation between HPV infection and tumorigenesis [5, 7, 23, 26]. In this study, the expression of HPV-16 E6 oncoprotein was determined by IHC analysis. The results of IHC analysis showed that the positive rate of E6 protein was as high as 47.44%, which was higher than all previous studies. The difference may be due to the higher false-negative rate of PCR. When HPV is eradicated after infection, the genomic DNA of HPV may not be detected by PCR. However, the sustained expression of E6 protein can be detected after HPV infection due to the integration of HPV E6 DNA sequence into the host genome [27]. In addition, the results of this study found that E6 expression was associated with good prognosis of UTUC but negatively correlated with T staging and LVI. This finding is inconsistent with other studies in bladder cancer [23, 26], which may be caused by different tumor types and small sample sizes.

We found APOBEC3B was highly expressed in UTUC tumors, which is consistent to other studies [8]. In addition, we found that the APOBEC3B was associated with good prognosis of UTUC and is negatively associated with UTUC T stage. However, studies on ovarian cancer and myeloma have shown that APOBEC3B expression is associated with poor prognosis [28, 29]. In contrast, our findings are supported by Glaser's study, which showed that APOBEC-mediated mutagenesis in urothelial carcinoma is associated with good prognosis [30].

Correlation analysis showed that APOBEC3B expression was positively correlated with E6 expression, which partially supported our hypothesis. In addition, APOBEC3B and E6 were both associated with low UTUC grade and staging, which explained their relationship with good prognosis. The phenomenon is similar to HPV-independent cervical cancer, which have lymph node involvement in the early stages, more distant metastasis, and generally worse oncological outcomes. That may be related to molecular typing and unique mechanism of tumorigenesis [31].

There are several limitations in this study. First, the sample size of this study is small. Second, there is a lack of basic research to logically support our finding that HPV infection can promote the occurrence of urothelial carcinoma by upregulating the expression of APOBEC3B and HPV E6 protein. Therefore, further basic research is needed to further support the findings of this study.

## 5. Conclusion

We examined the expression of HPV E6 and APOBEC3B in UTUC tissues through IHC analysis and explored the crucial roles of HPV E6 and APOBEC3B on the prognosis of UTUC. The results showed that E6 expression was associated with APOBEC3B expression, and both APOBEC3B and E6 were associated with good prognosis of UTUC. This study provides new evidence for further research on the relationship between HPV infection, APOBEC3B expression, and prognosis of UTUC.

## Figures and Tables

**Table 1:** Correlation between E6/APOBEC3B expression and clinical characteristics in 78 UTUC patients.

Clinic pathological features	All case	HPV E6 expression			APOBEC3B expression		
		Negative	Positive	*P* value	Low	High	*P* value
Median age, years (range)	70 (47-91)	72 (47-91)	70 (49-85)	0.434	72 (48-91)	69 (47-85)	0.564
*Sex,n(%)*							
Male	52 (66.67)	31 (39.74)	21 (26.92)	0.078	34 (43.59)	18 (23.08)	0.191
Female	26 (33.33)	10 (12.82)	16 (20.51)		13 (16.66)	13 (16.66)	
*Smoking,n(%)*							
Yes	33 (42.31)	19 (24.36)	14 (17.95)	0.448	20 (25.64)	13 (16.66)	0.956
No	45 (57.69)	22 (28.21)	23 (29.48)		27 (34.61)	18 (23.08)	
*Tumor location,n(%)*							
Calix or pelvis	47 (60.26)	27 (34.62)	20 (25.64)	0.288	31 (39.74)	16 (20.51)	0.205
Ureter	31 (39.74)	14 (17.95)	17 (21.79)		16 (20.51)	15 (19.23)	
*Pathological T stage,n(%)*							
pTa	10 (12.82)	2 (2.56)	8 (10.26)	**0.045**	5 (6.41)	5 (6.41)	0.267
pT1	23 (29.49)	10 (12.82)	13 (16.67)		12 (15.38)	11 (14.10)	
pT2	9 (11.54)	5 (6.41)	4 (5.13)		7 (8.97)	2 (2.56)	
pT3	21 (26.92)	12 (15.38)	9 (11.54)		11 (14.10)	10 (12.82)	
pT4	15 (19.23)	12 (15.38)	3 (3.85)		12 (15.38)	3 (3.85)	
*Pathological N stage,n(%)*							
pNx or pN0	73 (93.59)	37 (47.43)	36 (46.16)	0.420	43 (55.13)	30 (38.46)	0.645
pN1-2	5 (6.41)	4 (5.13)	1 (1.28)		4 (5.13)	1 (1.28)	
*Tumor stage AJCC staging,n(%)*							
I	32 (41.03)	12 (15.38)	20 (25.64)	**0.012**	15 (19.23)	17 (21.79)	**0.034**
II	8 (10.26)	2 (2.56)	6 (7.69)		3(3.85)	5 (6.41)	
III	21 (26.92)	14 (17.95)	7 (8.97)		15 (19.23)	6 (7.69)	
IV	17 (21.79)	13 (16.66)	4 (5.13)		14 (17.95)	3 (3.85)	
*Tumor grade,n(%)*							
Low	14 (17.95)	4 (5.13)	10 (12.82)	**0.047**	4 (5.13)	10 (12.82)	**0.007**
High	64 (82.05)	37 (47.43)	27 (34.62)		43 (55.13)	21 (26.92)	
*LVI,n(%)*							
Yes	16 (20.51)	12 (15.38)	4 (5.13)	**0.044**	11 (14.10)	5 (6.41)	0.436
No	62 (79.49)	29 (37.18)	33 (42.31)		36 (46.16)	26 (33.33)	

**Table 2:** Univariate and multivariate Cox regression analyses for DFS.

Variables	Univariate			Multivariate		
	HR	CI 95%	*P* value	HR	CI 95%	*P* value
Age < 70 vs.≥70	1.285	0.720-2.293	0.397			
Gender male vs. female	0.716	0.396-1.296	0.270			
Smoking yes vs. no	0.946	0.522-1.712	0.854			
LVI yes vs. no	2.103	1.102-4.015	0.024			
T STAGE pTa + pT1VS.pT2 + pT3 + pT4	2.346	1.242-4.431	0.009			
N STAGE pN0/Nx vs. pN+	3.241	1.144-9.182	0.027			
Grade high vs. low	1.207	0.561-2.597	0.631			
APOBEC3B	0.348	0.176-0.690	0.002	0.408	0.203-0.821	0.012
HPV E6	0.426	0.230-0.792	0.007			

**Table 3:** Correlation analysis of APOBEC3B, E6, and clinicopathological characteristics.

		APOBEC3B	HPV E6	LVI	T	N
APOBEC3B	*r*	1.000	**0.383** ^∗∗^	-0.088	**-0.251** ^∗^	-0.106
	*P*	/	0.001	0.439	0.028	0.354
E6	*r*	**0.383** ^∗∗^	1.000	**-0.228** ^∗^	**-0.225** ^∗^	-0.144
	*P*	0.001	/	0.045	0.048	0.207
LVI	*r*	-0.088	**-0.228** ^∗^	1.000	**0.413** ^∗∗^	0.126
	*P*	0.439	0.045	/	<0.001	0.268
pT	*r*	**-0.251** ^∗^	**-0.225** ^∗^	**0.413** ^∗∗^	1.000	0.213
	*P*	0.028	0.048	<0.001	/	0.062
pN	*r*	-0.106	-0.144	0.126	0.213	1.000
	*P*	0.354	0.207	0.268	0.062	/

## Data Availability

The data used to support the findings of this study are available from the corresponding author upon request.
